# Supplementary material for: Structure of the Dicer-2–R2D2 heterodimer bound to a small RNA duplex
Source: Nature. 2022 Jun 29;607(7918):393–8. doi: 10.1038/s41586-022-04790-2 (PMC9279153; doi:10.1038/s41586-022-04790-2)
Supplement: Supplementary file 1 — Uncropped images of gels and blots. [file 41586_2022_4790_MOESM1_ESM.pdf]

nature portfolio

https://doi.org/10.1038/s41586-022-04790-2

Supplementary information

Structure of the Dicer-2–R2D2 heterodimer bound to a small RNA duplex

In the format provided by the authors and unedited

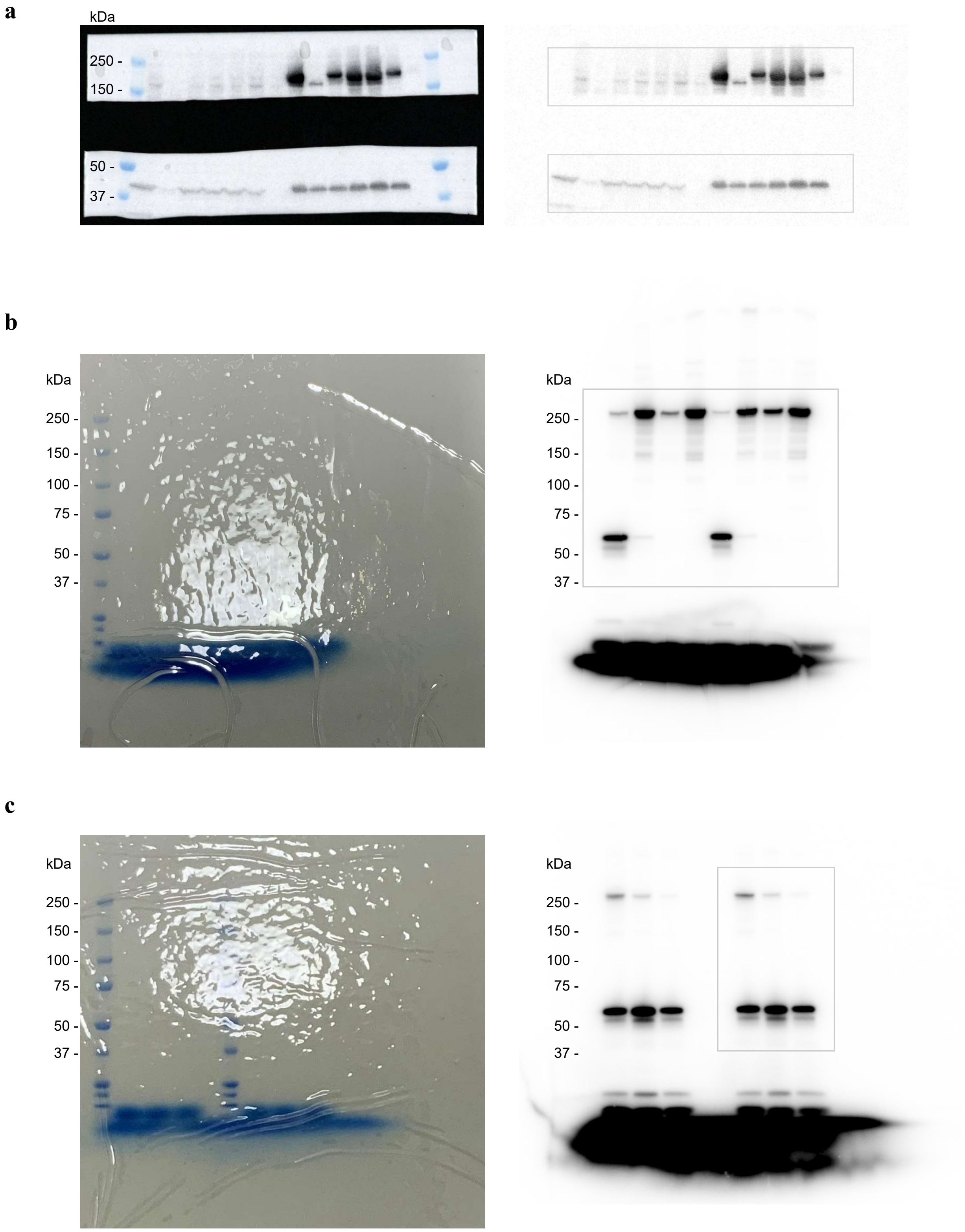

Supplementary Fig. 1. Uncropped images of gels and blots

a, Uncropped blot used for Fig. 2b.

b, Uncropped gel used for Fig. 4f.

c, Uncropped gel used for Extended Data Fig. 10c.
