# Supplementary material for: Structure of the Dicer-2–R2D2 heterodimer bound to a small RNA duplex
Source: Nature. 2022 Jun 29;607(7918):393–8. doi: 10.1038/s41586-022-04790-2 (PMC9279153; doi:10.1038/s41586-022-04790-2)
Supplement: Supplementary file 2 — Reporting Summary [file 41586_2022_4790_MOESM2_ESM.pdf]

nature portfolio

Corresponding author(s): Yukihide Tomari, Hiroshi Nishimasu, Osamu Nureki

Last updated by author(s): Mar 13, 2021

Reporting Summary

Nature Portfolio wishes to improve the reproducibility of the work that we publish. This form provides structure for consistency and transparency in reporting. For further information on Nature Portfolio policies, see our Editorial Policies and the Editorial Policy Checklist.

Statistics

For all statistical analyses, confirm that the following items are present in the figure legend, table legend, main text, or Methods section.

n/a	Confirmed	
☐	☒	The exact sample size (n) for each experimental group/condition, given as a discrete number and unit of measurement
☐	☒	A statement on whether measurements were taken from distinct samples or whether the same sample was measured repeatedly
☒	☐	The statistical test(s) used AND whether they are one- or two-sided<br>*Only common tests should be described solely by name; describe more complex techniques in the Methods section.*
☒	☐	A description of all covariates tested
☒	☐	A description of any assumptions or corrections, such as tests of normality and adjustment for multiple comparisons
☒	☐	A full description of the statistical parameters including central tendency (e.g. means) or other basic estimates (e.g. regression coefficient) AND variation (e.g. standard deviation) or associated estimates of uncertainty (e.g. confidence intervals)
☒	☐	For null hypothesis testing, the test statistic (e.g. F, t, r) with confidence intervals, effect sizes, degrees of freedom and P value noted<br>*Give P values as exact values whenever suitable.*
☒	☐	For Bayesian analysis, information on the choice of priors and Markov chain Monte Carlo settings
☒	☐	For hierarchical and complex designs, identification of the appropriate level for tests and full reporting of outcomes
☒	☐	Estimates of effect sizes (e.g. Cohen's d, Pearson's r), indicating how they were calculated

Our web collection on statistics for biologists contains articles on many of the points above.

Software and code

Policy information about availability of computer code

Data collection	Serial EM (version 3.7.10)
Data analysis	RELION (version 3.0 and version 3.1), Servalcat (version 0.2.0), COOT (version 0.9), UCSF ChimeraX (version 1.1.1), CueMol2 (http://www.cuemol.org/ version 2.2.3.443)

For manuscripts utilizing custom algorithms or software that are central to the research but not yet described in published literature, software must be made available to editors and reviewers. We strongly encourage code deposition in a community repository (e.g. GitHub). See the Nature Portfolio guidelines for submitting code & software for further information.

Data

Policy information about availability of data

All manuscripts must include a data availability statement. This statement should provide the following information, where applicable:

- Accession codes, unique identifiers, or web links for publicly available datasets
- A description of any restrictions on data availability
- For clinical datasets or third party data, please ensure that the statement adheres to our policy

The cryo-EM density maps and atomic coordinates have been deposited in the Electron Microscopy Data Bank. The accession codes for the maps are EMD-31741 (Dicer-2–R2D2) and EMD-31742 (Dicer-2–R2D2–siRNA). The accession codes for the coordinates are 7V6B (Dicer-2–R2D2) and 7V6C (Dicer-2–R2D2–siRNA).

Field-specific reporting

Please select the one below that is the best fit for your research. If you are not sure, read the appropriate sections before making your selection.

☒ Life sciences ☐ Behavioural & social sciences ☐ Ecological, evolutionary & environmental sciences

For a reference copy of the document with all sections, see nature.com/documents/nr-reporting-summary-flat.pdf

Life sciences study design

All studies must disclose on these points even when the disclosure is negative.

Sample size	For cryo-EM analyses, sample sizes were determined by the availability of microscope time and the number of particles on electron microscopy grids enough to obtain a structure at the reported resolution. For biochemical analysis, sample size were determined based on the previous reports of this type of study (Tomari et al., 2004) and the reproducibility of results across independent experiments.
Data exclusions	For cryo-EM analyses, particles that did not contribute to improving map quality were excluded following the standard classification procedures in RELION. This is standard practice for structure determination by cryo-EM. For biochemical analyses, no data was excluded.
Replication	For cryo-EM analyses, related experiments including purification, and SDS-PAGE were reproduced at least two times and structure determination was completed once based on the previous reports of this type of study. For biochemical analyses, all measurements were repeated at least three times. All attempts at replication were successful.
Randomization	For cryo-EM analyses, particles were randomly assigned to half-maps for resolution determination following the standard procedures in RELION. For biochemical analyses, randomization was not performed because this study does not involve animal experiments.
Blinding	Blinding is not applicable since this is an exploratory study and blinding is impossible or unlikely to affect the results or interpretation of the results.

Reporting for specific materials, systems and methods

We require information from authors about some types of materials, experimental systems and methods used in many studies. Here, indicate whether each material, system or method listed is relevant to your study. If you are not sure if a list item applies to your research, read the appropriate section before selecting a response.

Materials & experimental systems

n/a	Involved in the study	
☐	☒	Antibodies
☐	☒	Eukaryotic cell lines
☒	☐	Palaeontology and archaeology
☒	☐	Animals and other organisms
☒	☐	Human research participants
☒	☐	Clinical data
☒	☐	Dual use research of concern

Methods

n/a	Involved in the study	
☒	☐	ChIP-seq
☒	☐	Flow cytometry
☒	☐	MRI-based neuroimaging

Antibodies

Antibodies used	anti-FLAG-antibody (Sigma-Aldrich F3165, 1/2000)<br>anti-Dicer-2-antibody (Abcam ab4732, 1/1000)<br>Goat anti-mouse IgG (H+L)/(H&L) antibody, HRP conjugate (proteintech SA00001-1, 1/4000)<br>Goat anti-rabbit IgG (H+L)/(H&L) antibody, HRP conjugate (proteintech SA00001-2, 1/4000)
Validation	anti-FLAG-antibody (Sigma-Aldrich F3165) https://www.sigmaaldrich.com/specification-sheets/120/274/F3165-BULK.pdf<br>anti-Dicer-2-antibody (Abcam ab4732) https://www.abcam.co.jp/dcr-2--dicer-2-antibody-ab4732.html<br>Goat anti-mouse IgG (H+L)/(H&L) antibody, HRP conjugate https://www.ptglab.co.jp/products/HRP-conjugated-Affinipure-Goat-Anti-Mouse-IgG-H-L-secondary-antibody.htm<br>Goat anti-rabbit IgG (H+L)/(H&L) antibody, HRP conjugate https://www.ptglab.co.jp/products/HRP-conjugated-Affinipure-Goat-Anti-Rabbit-IgG-H-L-secondary-antibody.htm

Eukaryotic cell lines

Policy information about cell lines

Cell line source(s)	Sf9 (ATCC, Cat.#CRL-1711)
Authentication	sf9 cells were purchased from ATCC Cell lines and no further authentication was performed.
Mycoplasma contamination	sf9 cells were not tested for mycoplasma contamination.
Commonly misidentified lines (See ICLAC register)	sf9 cells are not misidentified cell lines.
