# Supplementary material for: Structure of the Dicer-2–R2D2 heterodimer bound to a small RNA duplex
Source: Nature. 2022 Jun 29;607(7918):393–8. doi: 10.1038/s41586-022-04790-2 (PMC9279153; doi:10.1038/s41586-022-04790-2)
Supplement: Supplementary file 4 — Data collection, processing, model refinement, and validation. [file 41586_2022_4790_MOESM4_ESM.pdf]

**Supplementary Table 1 | Data collection, processing, model refinement, and validation.**		
**Data collection and processing**		
Sample	Dicer-2–R2D2	Dicer-2–R2D2–siRNA
EMDB ID	EMD-31741	EMD-31742
PDB ID	7V6B	7V6C
Microscope	Titan Krios G3i	Titan Krios G3i
Detector	Gatan K3 camera	Gatan K3 camera
Magnification	105,000	105,000
Voltage (kV)	300	300
Electron exposure ($e^{-}/Å^2$)	53	48
Defocus range (μm)	−0.8 to −1.6	−0.8 to −1.6
Pixel size (Å)	0.83	0.83
Symmetry imposed	*C*1	*C*1
Initial particle images	1,699,210	2,181,396
Final particle images	144,979	179,826
Map resolution (Å)	3.3	3.3
FSC threshold	0.143	0.143
Map sharpening *B* factor ($Å^2$)	−95.85	−101.54
**Model building and refinement**		
Model composition		
Protein atoms	12,343	12,747
Nucleic acid atoms	0	1,099
R.m.s. deviations		
Bond lengths (Å)	0.0118	0.0118
Bond angles (°)	1.66	1.55
**Validation**		
MolProbity score	2.18	2.01
Clashscore	6.44	6.07
Rotamer outliers (%)	4.95	3.9
Ramachandran plot		
Favored (%)	95.73	96.55
Allowed (%)	4.27	3.45
Outliers (%)	0	0
