# Supplementary material for: Structure of the Dicer-2–R2D2 heterodimer bound to a small RNA duplex
Source: Nature. 2022 Jun 29;607(7918):393–8. doi: 10.1038/s41586-022-04790-2 (PMC9279153; doi:10.1038/s41586-022-04790-2)
Supplement: Supplementary file 5 — Nucleic acid sequences used in this study. [file 41586_2022_4790_MOESM5_ESM.pdf]

Supplementary Table 2 | Nucleic acid sequences used in this study.		
**Oligonucleotides used for plasmid construction**		
Construct	Forward primer	Reverse primer
pFastBac_NGFP_Dicer-2	TACTTTCAGGGCTCTATGGAA	CAGCTAAGCAAATGCGACGCCTAAG
pFastBac_NGFP_R2D2	CCGGTCCGAAGCGCGGCCACC	CGACCATATAATAGACTATTTTCGCAC
pFastBac_His_Dicer-2	ATGGAAGATGTGGAAATCAAGCC	GTGATGGTGATGGTGATGGGTGGCCG
pFastBac_vector	GAGGATCATAATCAGCCATACCAC	GGAGAAAACCTGTACTTTCAGGGCTCT
**Oligonucleotides used for structural determination**		
Guide	UGAGGUAGUAGGUUGUAUAGU	
Passenger	UAUACAACCUACUACCUCUCU	
**Oligonucleotides used for structural determination**		
Mutation	Forward primer	Reverse primer
ΔCL	GGCGGCGGCAGTCCAGCGGAGCA	AGGCAGCAGATGGAGGTTTAAATACG
Δ1081-1116	GGTAATGTATGTGAGATGAATGAGAT	TTCTGTTATTTCAAGATTGGCCACGGAC
R324E	GAGACGGCCTTGACCTTGTGCGAGA	ATGCATCAATTTCACGCTTAGCGTCTC
E331R	GAGGATCATAATCAGCCATACCAC	GGAGAAAACCTGTACTTTCAGGGCTCT
R324E/E331R	AAAATCCGTCACTTGCTGGTGCAAA	GCGGCACAAGGTCAAGGCCGTCTCATG
**Oligonucleotides used for the R2D2 mutant preparation (pFB-3×FLAG-R2D2)**		
Mutation	Forward primer	Reverse primer
W205A	GAGTTGAAGGAAGCCGGGAGCGTA	GGCGTAGGTGGTAAATTTCTTTCTGCG
K98A	ATGGTGGCCGAGCTGCGCGACTAC	CTAAACCGGGACATGGTGGCCGAGCTG
ΔCTD	GGAGGAGGATCGCATACAGGCATG	AAATTTCTTTCTGCGCAATGCCTCCAAT
**Oligonucleotides used for structural determination**		
siRNA-1	UGAGGUAGUAGGUUGUAUAαU	α = 5-I-U
siRNA-2	UAUACAACCUACUACCUCUUU	
siRNA-3	AAUACAACCUACUACCUCAUU	
siRNA-4	UGAGGUAGUAGGUUGUAUAα	α = 5-I-U
siRNA-5	AUAUACAACCUACUACCUCUUU	
